# Laparoscopic and endoscopic co-operative surgery for a non-ampullary duodenal tumor after living-donor liver transplantation: a case report

**DOI:** 10.1093/jscr/rjaf023

**Published:** 2025-01-22

**Authors:** Shunichi Ito, Rinka Tamazaki, Shinsuke Maeda, Kei Hosoda

**Affiliations:** Department of Surgery, Institute of Gastroenterology, Tokyo Women’s Medical University, 8-1, Kawadacho, Shinjuku-ku, Tokyo 162-8666, Japan; Department of Surgery, Institute of Gastroenterology, Tokyo Women’s Medical University, 8-1, Kawadacho, Shinjuku-ku, Tokyo 162-8666, Japan; Department of Surgery, Institute of Gastroenterology, Tokyo Women’s Medical University, 8-1, Kawadacho, Shinjuku-ku, Tokyo 162-8666, Japan; Department of Surgery, Institute of Gastroenterology, Tokyo Women’s Medical University, 8-1, Kawadacho, Shinjuku-ku, Tokyo 162-8666, Japan

**Keywords:** D-LECS, living donor liver transplantation, non-ampullary duodenal tumor

## Abstract

Superficial duodenal epithelial tumors were previously considered rare. Laparoscopic and endoscopic cooperative surgery for duodenal tumors (D-LECS) has been developed to achieve successful endoscopic treatment. Patients who have undergone living-donor liver transplantation (LDLT) may have severe abdominal adhesions, and immunosuppressive agents (IAs) may affect the degree of postoperative abdominal adhesions and wound healing, but their effects remain unclear. Herein, we present the first case of D-LECS for duodenal adenoma after LDLT. A 66-year-old man underwent D-LECS for a non-ampullary duodenal high-grade adenoma after LDLT with an IA. The patient’s condition was uneventful 36 months after the surgery. In gastrointestinal surgery, IAs may affect the resected duodenal repair process. For duodenal neoplasms in high-risk patients, D-LECS may be better than endoscopic submucosal dissection alone. D-LECS after LDLT is a feasible and less invasive procedure.

## Introduction

The capacity to detect a superficial duodenal epithelial neoplasm has increased because of the development of endoscopic instruments [1, 2].

In particular, for patients with low-risk duodenal neoplasms (DNs), which have a lower risk of lymph node metastasis, conventional surgery such as pancreatoduodenectomy remains invasive [2]. The least invasive procedure for non-ampullary DNs is endoscopic resection (ER) [3, 4].

Laparoscopic and endoscopic cooperative surgery (LECS) for the gastric submucosal tumors, and LECS for DNs (D-LECS) were developed to enable successful endoscopic treatment of neoplasms by avoiding perforation during and after endoscopic submucosal dissection (ESD) [5–7]. D-LECS can prevent adverse events such as perforation and bleeding with additional laparoscopic suturing at the ESD site, minimizing duodenal distortion [2].

Generally, in patients receiving immunosuppressive agents (IAs), peritoneal adhesions may be mild. However, patients who have undergone living-donor liver transplantation (LDLT) may have severe abdominal adhesions, and IAs may affect the degree of postoperative abdominal adhesions and wound healing, but their effects remain unclear [8]. Here, we present a case of partial duodenal resection with D-LECS for duodenal adenoma after LDLT.

## Case report

A 66-year-old man underwent LDLT with a right lobe graft from his second son for liver failure due to polycystic liver and kidney disease 6 years previously. He has continued to take IAs (tacrolimus and mycophenolate mofetil) since transplantation. During a follow-up, a duodenal tumor was detected on upper gastrointestinal endoscopy.

**Figure 1:**
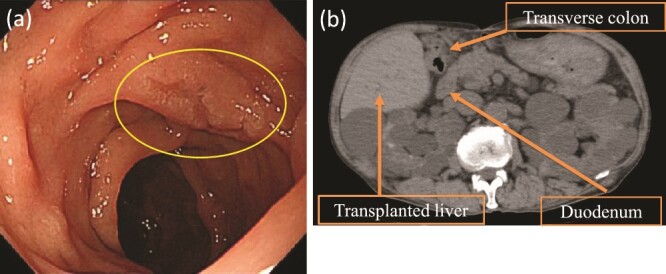
Upper gastrointestinal endoscopy and abdominal plain CT findings. (a) Upper gastrointestinal endoscopy showing the elevated lesion (10 mm in diameter) at the opposite side of the ampulla of Vater. (b) CT showing the transplanted liver very close to the transverse colon (axial image).

Upper gastrointestinal endoscopy revealed a high-grade adenoma by biopsy, 15 mm in diameter, on the opposite side of the ampulla of Vater (Fig. 1a), and we decided to perform D-LECS. Preoperative computed tomography (CT) revealed no metastasis to any other organs but that the transplanted liver was very close to the transverse colon (Fig. 1b). Moreover, strong adhesions around the liver were expected because of bile duct anastomotic leakage after LDLT. To avoid damage to the vital liver and transverse colon, we first considered to dissect the mesentery of the transverse colon before an approach from the anterior surface of the pancreatic head to the second portion of the duodenum to create an adequate space for surgery.

**Figure 2:**
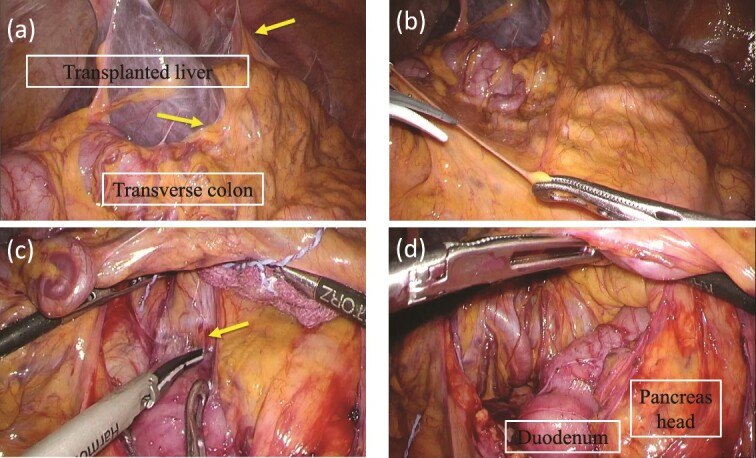
Intraoperative findings before ESD. (a) The liver adheres to the upper abdominal wall and transverse colon as expected (arrow). (b) The transvers mesentery is dissected. (c) There is strong adhesion around the duodenal bulb because of the previous surgery (arrow). (d) An adequate space around the tumor at the second portion of the duodenum is made.

**Figure 3:**
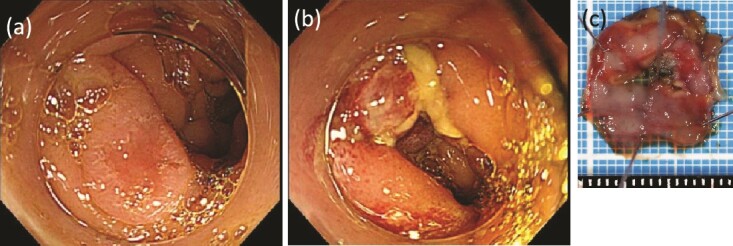
ESD was performed. (a) Before dissection. (b) After dissection. (c) The resected specimen.

**Figure 4:**
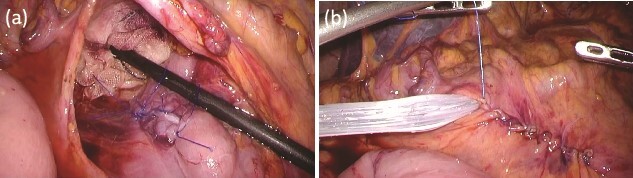
Intraoperative findings after ESD. (a) The seromuscular layer of the mucosal defect lesion is sutured laparoscopically to reinforce it. (b) After confirming sufficient reinforcement of the defect and no bleeding nor passage obstruction by both upper gastrointestinal endoscopy and laparoscopy, the transverse colon mesentery is closed to finish the operation.

While continuing to take IAs, the patient underwent D-LECS as planned. The first trocar was placed at the umbilicus level. Four other trocars were then inserted: one 12-mm trocar in the right lower quadrant and three 5-mm trocars in the right upper, left upper, and left lower quadrants. Intraoperatively, the liver adhered to the upper abdominal wall and transverse colon as expected (Fig. 2a). When the transverse mesentery was dissected to avoid injury to the colonic vessels (Fig. 2b), the second portion of the duodenum was approached, where strong adhesions were observed (Fig. 2c). An adequate space was created around the tumor (Fig. 2d). Next, ESD was performed (Fig. 3a and b), and the resected specimen was retrieved orally. Next, the seromuscular layer of the mucosal defect lesion was sutured laparoscopically to reinforce it because it was endoscopically difficult to close the mucosal defect at the post-ESD site (Fig. 4a). After confirming sufficient reinforcement of the defect and no bleeding nor passage obstruction by both upper gastrointestinal endoscopy and laparoscopy, the transverse colon mesentery was closed to finish the operation (Fig. 4b). The operative time was 282 min, and the intraoperative blood loss was 10 mL. Microscopically, the resected specimen was a high-grade tubular adenoma with negative surgical margins (Fig. 3c). The patient was discharged on postoperative day 9 uneventfully. No postoperative therapy was administered, and the patient’s condition was uneventful 38 months after surgery.

## Discussion

In general, the non-ampullary duodenal adenoma ≤20 mm is an indication of ER, such as cold polypectomy, endoscopic mucosal resection (EMR), and underwater EMR [9]. In our case, the tumor was small; however, postoperative adhesions might have led to technical inability in ER only because it was not possible to approach the tumor stably. First, we performed adhesion detachment by laparoscopy to be able to perform ER with laparoscopic assistance. Next, the tumor could be brought closer so we tried to perform EMR or under EMR, but we just couldn’t seem to do en bloc resection. As a result, we selected ESD to perform en bloc resection.

In ESD for non-ampullary DNs, the complication rate is high [3]. To manage the perforations associated with duodenal ESD, a complete closure of the entire mucosal defect is endoscopically important [10]. Moreover, suturing the seromuscular layer at the ESD site is necessary to prevent postoperative leakage [2]. Factors complicating ESD, such as a larger tumor size or longer operation time, might be associated with perforation [10]. On the other hand, IAs may affect the degree of postoperative abdominal adhesions and wound healing, but their effects on the other aspects remain unclear [8]. In gastrointestinal surgery, IAs may affect the resected duodenal repair process. For DNs in high-risk patients, such as those with an immunosuppressive state or suspected postoperative adhesions around the duodenum, it may be better to choose D-LECS than ESD alone.

For D-LECS, it reported two distinct approaches according to tumor location; the antecolic approach was available for a wide range of DNs but required several complicated procedures and entailed a risk of duodenal wall injury [3]. In contrast, the retrocolic approach is available and effective for DNs located at the anal side of the papilla of Vater in the descending and horizontal portions. In this case, the tumor was located on the opposite side of the ampulla of Vater; however, the transverse colon was expected to adhere to the transplanted liver preoperatively. It reported that all patients after LDLT had severe abdominal adhesions, whereas peritoneal adhesions were mild in patients receiving IAs [8]. Therefore, we selected the retrocolic approach, and we were able to approach the second portion of the duodenum without damaging the liver and colon and safely perform D-LECS. Therefore, these two approaches can be selected according to the tumor location and degree of adhesion around the duodenum preoperatively and intraoperatively.

In conclusion, D-LECS after LDLT is a feasible and less invasive procedure.
